# Lower limb motor effects of DBS neurofeedback in Parkinson’s disease assessed through IMU-based UPDRS movement quality metrics

**DOI:** 10.1038/s41598-025-28378-8

**Published:** 2025-11-27

**Authors:** Lena Salzmann, Oliver Bichsel, Manabu Rohr-Fukuma, Aileen C. Naef, Lennart Stieglitz, Markus F. Oertel, Bartosz Bujan, Piotr Jedrysiak, Olivier Lambercy, Lukas L. Imbach, Roger Gassert

**Affiliations:** 1 Rehabilitation Engineering Laboratory, Department of Health Sciences and Technology, ETH Zurich, 8092 Zurich, Switzerland; 2 Department of Neurosurgery, University Hospital Zurich, University of Zurich, 8091 Zurich, Switzerland; 3 Clinical Neuroscience Centre, University Hospital Zurich, University of Zurich, 8091 Zurich, Switzerland; 4 Data Analytics & Rehabilitation Technology, Lake Lucerne Institute, 6354 Vitznau, Switzerland; 5 Neurorehabilitation, Klinik Lengg, 8008 Zurich, Switzerland; 6 Swiss Epilepsy Center, Klinik Lengg, 8008 Zurich, Switzerland; 7 Institute for Neuroscience, Department of Health Sciences and Technology, ETH Zurich, Zurich, Switzerland; 8 The LOOP Zurich – Medical Research Center, Zurich, Switzerland

**Keywords:** Parkinson’s disease, Motor function assessment, Wearable sensors, Neurorehabilitation, Neurofeedback, Beta oscillations, Motor control, Parkinson's disease, Translational research

## Abstract

Parkinson’s disease (PD) is characterized by progressive motor impairments, including lower limb dysfunction, leading to reduced mobility and increased fall risk. To counteract these deficits, neurofeedback based on deep brain stimulation (DBS) electrodes has been proposed as a novel approach to mitigate motor symptoms via modulation of abnormal beta-oscillations in the subthalamic nucleus. However, its potential to improve motor symptoms has yet to be fully established. This study examined whether a single session of DBS-based neurofeedback could have a short term effect on movement quality, quantified through inertial measurement unit recordings. Ten PD patients performed two standardized motor tasks, foot stomping and hand pronation-supination, from the Unified Parkinson’s Disease Rating Scale. Movement quality metrics from inertial measurement units were extracted and compared before and after neurofeedback-induced beta-power downregulation. Beta-power was successfully reduced by -12.42% on average, and the reduction was associated with significant improvements in lower limb movement quality metrics—acceleration magnitude (p = 0.037), movement speed (steps per second: p = 0.010; mean peak velocity: p = 0.002), and reduced halts (p = 0.020)—with a strong coupling between beta reduction and speed gain (Spearman $\rho$ = 0.976, p < 0.001). No significant improvements were observed in upper limb movements. These findings indicate that neurofeedback-driven downregulation of beta-power produces measurable enhancements in lower limb movement quality, captured through wearable sensor metrics. Future work should assess whether these improvements translate into lasting functional benefits and validate the clinical relevance of these metrics.

## Introduction

Parkinson’s disease (PD) is a prevalent neurodegenerative disorder that primarily affects motor function, leading to symptoms such as tremor, bradykinesia, rigor, and postural instability^1,2^. These motor symptoms are linked to the degeneration of dopaminergic neurons in the substantia nigra, and disrupted neural activity within the basal ganglia-thalamo-cortical circuit. A key consequence of this disruption is excessive synchronization and elevated power in the beta frequency band (13–30 Hz) within the subthalamic nucleus (STN), which has been shown to correlate with the severity of motor impairments in PD patients^3–6^.

To counteract these abnormal neural oscillations, current standard treatments include dopamine replacement medication, such as levodopa, and deep brain stimulation (DBS) of the STN. Both therapies aim to modulate the abnormal beta-power and improve motor performance^7–11^. However, these effects are often temporary and show significant variability across patients. In particular, gait disturbances and postural instability are frequently resistant to treatment and may even worsen over time^12–14^. In some cases, the interventions may even increase specific motor symptoms or lead to adverse effects^15,16^, underscoring the need for complementary therapeutic strategies.

Neurofeedback has been proposed as a novel, non-pharmacological adjunct therapy for PD^17^. It is a technique that trains individuals to self-regulate neural activity by providing real-time feedback on specific brain signals, enabling them to modulate abnormal activity at will. This approach has been explored using various modalities, including electroencephalography and functional MRI, in both healthy individuals and clinical populations^18–20^. In the context of PD, DBS-based neurofeedback offers direct access to STN local field potentials (LFPs), allowing the real-time extraction and visualization of beta-power. Patients can learn to downregulate this beta-power through visual neurofeedback, as demonstrated in recent studies^21–23^. The downregulation of beta-power via neurofeedback has been associated with improvements in motor initiation^15^ and faster forearm movements^24^.

Despite these promising early results, neurofeedback interventions often use beta-power reduction as an indirect proxy for therapeutic success or rely on clinician-administered motor assessments that are inherently subjective and prone to inter-rater variability. While these clinical scales such as the Movement Disorder Society - Unified Parkinson’s Disease Rating Scale, part III (MDS-UPDRS III)^25^ remain widely used, they are limited in their temporal resolution and objectivity. This presents two critical challenges: first, changes in neural oscillations may not directly reflect meaningful motor improvements; second, manual evaluations lack the reproducibility required for broader clinical adoption and may be insensitive to the subtle changes in movement quality that neurofeedback may induce.

To bridge this gap, sensitive and objective tools are needed to assess whether neurofeedback-induced neural changes lead to functional motor improvements. Wearable inertial measurement units (IMUs) provide a promising alternative, enabling high-resolution, quantitative tracking of motor performance features such as acceleration, velocity, and smoothness. IMUs have been used extensively in PD research^26–29^, demonstrating their ability to capture changes in motor tasks that often remain undetected by human raters. Despite the growing use of IMUs and the increasing evidence on DBS-based neurofeedback, their combined application to systematically evaluate the effects of neurofeedback on movement quality remains scarce. To our knowledge, only one study has directly addressed this question in PD^24^, highlighting the need for further systematic investigation. Moreover, the one-by- one replication of MDS-UPDRS III subitems such as speed and amplitude has only been addressed in few studies, which are either not taking the MDS-UPDRS III as a direct template^30^, are relying on non-interpretable methods like machine learning^31,32^, or are focusing on the general use of wearable sensors to measure gait parameters or improve management of symptoms^33^.

The present study investigates the short-term effects of single-session DBS-based neurofeedback on movement quality in individuals with PD. Two standardized MDS-UPDRS III motor tasks - hand pronation-supination (HPS) and seated foot stomping (FS) - were selected because they can be reliably performed in a controlled laboratory setting and are highly sensitive to subtle motor changes. Movement quality was assessed using task-specific, computationally derived IMU metrics before and after one minute neurofeedback for beta-downregulation. We hypothesize that voluntary beta-power downregulation is feasible and associated with measurable improvements in movement quality. These findings aim to inform the design of neurofeedback protocols and encourage using objective metrics from wearables in movement analysis.

## Results

Ten participants with idiopathic PD (mean age ± SD = 60±12 years; four female) completed the study protocol without adverse events. All participants underwent a single neurofeedback session to downregulate subthalamic beta-power, followed by movement quality assessments based on IMU recordings.

### Neurofeedback

#### Beta-power modulation

**Fig. 1:**
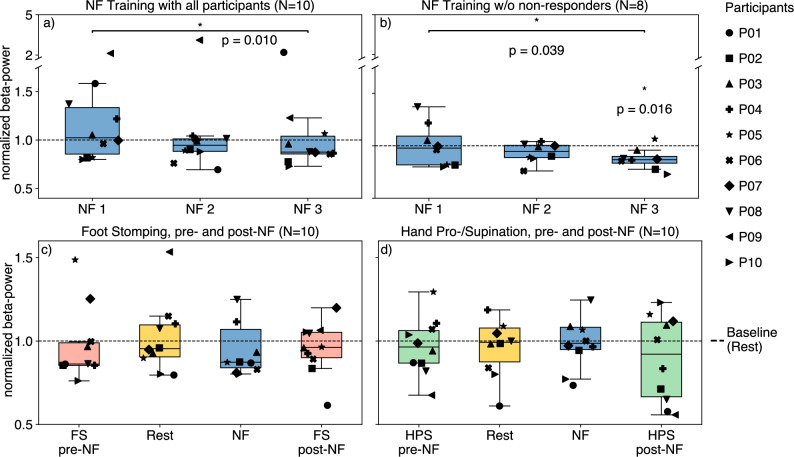
Median beta-power during the recording blocks. For each participant, the beta-power value was normalized by the median baseline rest period of each block. (**a**) Neurofeedback training blocks across all participants; (**b**) Neurofeedback training blocks without non-responders. The p-value and significance star on the right (p = 0.016) are indicating the difference of the third neurofeedback training block compared to baseline; (**c**) FS task block; (**d**) HPS task block. **NF:** Neurofeedback; **FS:** Foot Stomping; **HPS:** Hand pronation-supination.

Across three one-minute neurofeedback trainings, participants achieved an average reduction in beta-power of −12.42%, with a maximum observed reduction of −30.59% by using motor imagery strategies. Most of the strategies were related to motor tasks (e.g., imagined hand movements or walking), though some participants used non-motor strategies such as music or meditation (see Table 3). Fig. 1a shows baseline-normalized median beta-power across neurofeedback training blocks. A significant reduction in median beta-power was found between the first and third neurofeedback training block (W = 3.0, p = 0.039). After excluding two non-responders (P01 and P09), who could not achieve significant downregulation, this effect was more pronounced (W = 1.0, p = 0.016; Fig. 1b, N=8). For reference, median beta-power reductions during the three consecutive neurofeedback training blocks were +2.31%, –5.42%, and –12.42%, respectively.

Beta-power was also analyzed during the session blocks combining neurofeedback with FS and HPS motor tasks. In the FS block (Fig. 1c), the median beta-power change relative to the baseline was –13.75% during the pre-neurofeedback motor task, –12.67% during neurofeedback, –3.83% during the post-neurofeedback task, and –4.62% during rest. In the HPS block (Fig. 1d), the corresponding changes were smaller: –3.60% during the pre-neurofeedback task, –1.38% during neurofeedback, –7.90% during the post-neurofeedback task, and –0.76% during rest. No significant suppression of beta-power was observed in either block compared to the baseline rest period. In several cases, beta-power returned to baseline or increased, particularly during the HPS block. The same was done for responder only, but results remained unchanged (see n Material Fig. S1).

#### Spectral analysis

**Fig. 2:**
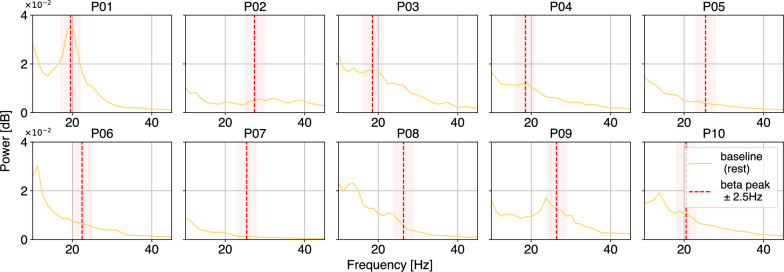
Power frequency spectra of the baseline recording for each of the study participants. To validate the accuracy of the selected beta-peak in the Medtronic BrainSense™software, the power-frequency-spectrum was plotted for the 60-second baseline rest recording of the first block for each participant. The individual beta-peak is indicated with the dashed red line, and the red-shaded area indicates the frequency range (beta-peak ± 2.5 Hz) displayed in the neurofeedback visualization.

**Fig. 3:**
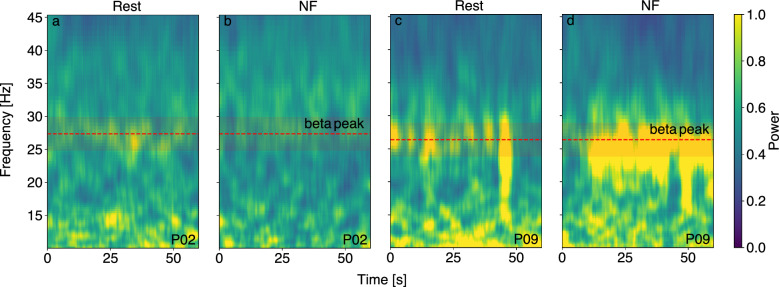
Time-frequency representation of power values. (**a** & **b**): example of a responder (P02). Compared to the baseline rest (**a**), the power at the beta-peak (red dashed line) appears decreased during the neurofeedback downregulation task (**b**) indicated by less power (orange coloring) in the area of the beta-peak. (**c** & **d**): example of a non-responding participant. The beta-power during the neurofeedback task was increased (**d**) compared to baseline (**c**). **NF:** Neurofeedback.

By plotting power spectra during the baseline rest period (Fig. 2), the beta-peak identification in the Medtronic BrainSense™software was validated. A slight overestimation of beta-peak frequency was observed (mean offset ± standard deviation over all participants in baseline rest condition: 1.37±1.97 Hz). Time-frequency representations (TFRs, Fig. 3) illustrate beta-power modulation: compared to baseline (Fig. 3a), participant P02, a responder to neurofeedback, exhibited a clear reduction in power around the beta-peak during downregulation (Fig. 3b). In contrast, the beta-power of participant P09, a non-responder, increased relative to the baseline. These individual patterns are consistent with the variability observed in the group-level beta-power statistics.

### IMU-derived movement quality metrics

To address potential test-retest effects, comparison of baseline and pre-neurofeedback motor tasks revealed significant differences in three variables: FS mean peak velocity (W = 7.0, p = 0.037) and FS acceleration magnitude (W = 2.0, p = 0.006), as well as HPS turn intervals (W = 5.0, p = 0.039). All three measures were worse at pre-neurofeedback compared to baseline.

*Lower Limb (FS):* Post-neurofeedback, significant improvements in movement quality were observed in FS performance. In the all-participants analysis (N=10), both speed metrics improved (steps per second: +5.7%, W = 3.0, p = 0.010; mean peak velocity: +36.1%, W = 0.0, p = 0.002), halts were reduced, as indicated by shorter step intervals (–4.5%, W = 5.0, p = 0.020), and acceleration magnitude increased (+14.5%, W = 7.0, p = 0.037).

**Fig. 4:**
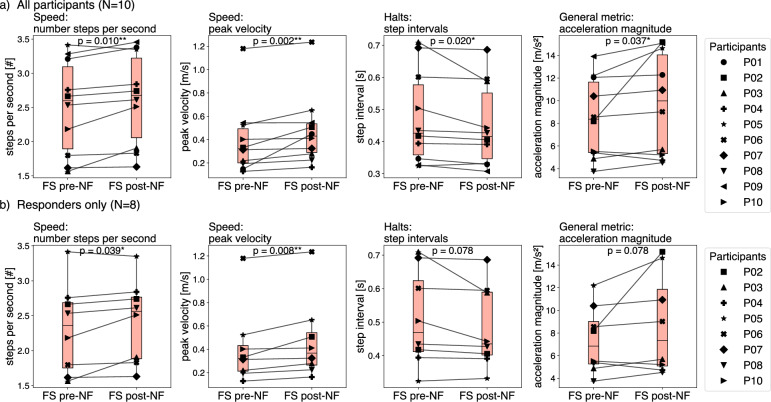
Pre- vs post-neurofeedback changes in foot-stomping (FS) performance. (**a**) All participants (N=10). (**b**) Responders only (N=8). Wilcoxon signed-rank tests indicated significant improvements in steps per second, peak velocity, and acceleration magnitude, as well as reduced step intervals in the all-participants analysis. In the responder-only analysis, improvements in both speed metrics remained significant, whereas step interval and acceleration magnitude showed the same trend but did not reach significance. **FS:** Foot Stomping, **NF:** Neurofeedback.

In the responders-only analysis (N=8), the same four metrics showed changes in the same direction as in the all participants analysis. Speed metrics remained significant (steps per second: W = 3.0, p = 0.039; mean peak velocity: W = 0.0, p = 0.008), whereas step interval (W = 5.0, p = 0.078) and acceleration magnitude (W = 5.0, p = 0.078) no longer reached the significance threshold. Sensitivity analyses excluding each non-responder individually (P01 or P09, N=9) demonstrated that all four metrics remained significant, confirming that the findings were not driven by an individual participant. Only when both P01 and P09 were excluded simultaneously (N=8) did two metrics lose statistical significance, though effect directions were unchanged. The results are illustrated in Fig. 4, with exact p-values and effect sizes reported in Table 1.

**Table 1:** Sensitivity analyses of foot-stomping metrics pre- vs post-neurofeedback. Wilcoxon signed-rank tests were conducted for (i) all participants (N=10), (ii) responders only (N=8), and after excluding non-responders individually (P01 or P09, N=9). Effect directions were consistent across analyses. All metrics remained significant when excluding P01, whereas exclusion of P09 led to a loss of significance for acceleration magnitude (p = 0.074). The reduction in significance in smaller subsets reflects decreased statistical power rather than reversal of the observed effects.

**Metric**	**All (N=10)**	**Responders (N=8)**	**Excl. P01 (N=9)**	**Excl. P09 (N=9)**
Steps per second	+5.71%; W = 3.0, p = 0.010**	W = 3.0, p = 0.039*	W = 4.0, p = 0.020*	W = 4.0, p = 0.020*
Mean peak velocity	+36.07%; W = 0.0, p = 0.002**	W = 0.0, p = 0.008**	W = 1.0, p = 0.004**	W = 1.0, p = 0.004**
Step interval	–4.54%; W = 5.0, p = 0.020*	W = 5.0, p = 0.078	W = 7.0, p = 0.039*	W = 7.0, p = 0.039*
Acceleration magnitude	+14.51%; W = 7.0, p = 0.037*	W = 5.0, p = 0.078	W = 8.0, p = 0.039*	W = 10.0, p = 0.074

*Upper Limb (HPS):* No significant changes were detected for HPS metrics when comparing pre-neurofeedback to post-neurofeedback executions. Angular velocity and movement timing showed variability, but differences between pre- and post-neurofeedback did not reach statistical significance (Table 2). Consistent with the non-significant Wilcoxon tests for HPS, a paired equivalence test indicated that the HPS mean-peak-velocity change was statistically equivalent to no meaningful effect ($p_{\max }=0.006$, $N=10$).

**Table 2:** Wilcoxon test results and effect sizes (SMD with 95% CI) for pre- vs. post-neurofeedback movement metrics. **FS:** Foot Stomping, **HPS:** Hand Pronation Supination, **SMD:** Standardized Mean Difference, $W$: test statistic; *$p < 0.05$, **$p < 0.01$.

**FS Metric (N=10)**	**W**	**p**	**SMD [95% CI]**	**HPS Metric (N=10)**	**W**	**p**	**SMD [95% CI]**
General Metrics							
Acceleration magnitude	7.0	0.037*	0.57 [–0.15, 1.28]	Angular velocity magnitude	26	0.092	0.20 [–0.51, 0.92]
Speed							
Steps per second	3.0	0.010**	0.94 [0.22, 1.65]	Turns per second	14.0	0.193	0.46 [–0.25, 1.18]
Mean peak velocity	0.0	0.002**	0.85 [0.13, 1.56]	Mean peak angular velocity	17.0	0.322	0.41 [–0.30, 1.13]
Amplitude							
Mean step amplitude	17.0	0.570	−0.02 [–0.74, 0.70]	Mean turn amplitude	24.0	0.770	−0.08 [–0.80, 0.63]
Total distance	27	1.000	0.02 [–0.70, 0.74]	Cumulative angular displacement	14	0.193	0.45 [–0.27, 1.16]
Hesitations							
Spectral arc length	12.0	0.131	0.41 [–0.30, 1.13]	Spectral arc length	23.0	0.695	0.08 [–0.64, 0.79]
Log-jerk velocity	9.0	0.064	0.62 [–0.10, 1.33]	Log-jerk velocity	17.0	0.322	−0.38 [–1.10, 0.34]
Halts							
Number of halts	0.0	0.059	0.71 [–0.01, 1.42]	Number of halts	2.5	0.625	−0.32 [–1.03, 0.40]
Step intervals	5.0	0.020*	−0.64 [–1.35, 0.08]	Turn intervals	12.0	0.131	−0.58 [–1.29, 0.14]
Decrementing Amplitude							
Amplitude change	17.0	0.322	0.04 [–0.68, 0.75]	Turn amplitude change	25.0	0.846	0.10 [–0.62, 0.81]
Slope of step height	20.0	0.492	−0.10 [–0.82, 0.61]	Slope of turn angle	24.0	0.770	−0.16 [–0.88, 0.55]

### Correlation between beta-power and movement quality

**Fig. 5:**
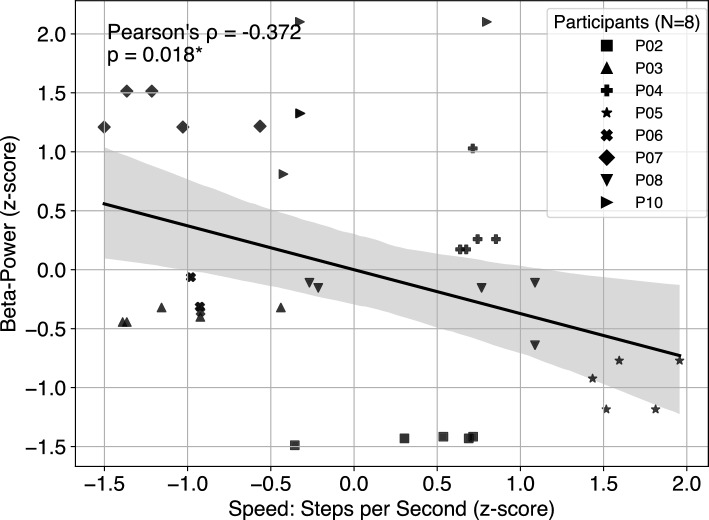
Correlation between beta-power and foot stomping speed (steps per second), across study session, excluding two non-responders (P01 and P09, N=8) to neurofeedback. Each participant contributed five data points: one during baseline foot stomping (FS), two during pre-neurofeedback FS, and two during post-neurofeedback FS. Both beta-power and movement speed were z-scored across all data points to allow comparison across participants and to report standardized effect sizes. The black regression line represents a linear fit across all trials, with a 95% confidence interval shown in gray.

No significant associations were found when all participants were included in the analysis. However, after excluding the two non-responders (P01 and P09), a moderate negative correlation emerged between beta-power and movement speed (steps per second, Pearson’s $\rho$ = −0.37, p = 0.018). Linear regression confirmed that beta-power significantly predicted movement performance (R^2^ = 0.14, F(1, 38) = 6.11, beta = −0.373, 95% CI [−0.686, −0.061], p = 0.018, N=40). Adding the task (baseline, pre-neurofeedback, post-neurofeedback) did not significantly improve model fit, and interaction terms between beta-power and task were non-significant. The negative association between beta-power and speed remained robust across FS conditions, indicating a consistent relationship between elevated beta activity and reduced motor performance (see Fig. 5).

At baseline, beta-power and FS speed showed the expected negative association (Spearman $\rho = -0.381$, 95% CI [–0.866, 0.615], $p = 0.352$, $N=8$), consistent in direction with established physiology, although not significant in this small sample. A change-score analysis revealed that larger beta reductions (pre–post) predicted larger speed gains (post–pre) across participants (Spearman $\rho = 0.976$, 95% CI [0.795, 1.000], $p < 0.001$, $N=8$). The same strong association was observed when restricting the analysis to responders only.

Limb-specific exploratory analyses did not reveal significant associations between baseline impairment and the ability to downregulate beta-power. For FS, correlations with MDS-UPDRS item 3.8 (measured side) were negative but non-significant (OFF: $\rho = -0.400, p = 0.251$; ON: $\rho = -0.342, p = 0.333$, n = 10). For HPS, a similar non-significant negative trend was observed in the OFF state ($\rho = -0.483, p = 0.157$), whereas the ON state showed no relationship ($\rho = 0.105, p = 0.773$, n = 10).

## Discussion

This study investigated the short-term effects of DBS-based neurofeedback on motor function in individuals with PD, focusing on movement quality quantified through IMU recordings. We demonstrated that participants could learn to downregulate pathological beta-power in the STN within a single neurofeedback session and that this neural modulation was associated with measurable improvements in lower limb motor performance. To our knowledge, this is the first study to assess neurofeedback-induced motor changes using MDS-UPDRS-aligned IMU metrics in both upper and lower limb tasks.

Eight out of ten participants achieved successful beta-power downregulation during the dedicated neurofeedback training blocks, with a group average reduction of −12.42%. This confirms prior findings that patients with PD can learn to volitionally suppress subthalamic beta-power with real-time feedback^21–23^. Two participants did not achieve successful downregulation and were classified as non-responders, a common occurrence in neurofeedback protocols^34,35^. Several factors may account for such non-responsiveness, including interindividual variability in cognitive strategies, differences in attentional capacity, or underlying neural dynamics that are less amenable to short-term modulation. To account for this variability in neurofeedback responsiveness, two complementary analysis sets were prespecified: a conservative all-participants analysis (N = 10) and a responders-only analysis (N = 8; non-responders P01 and P09 excluded). This dual reporting strategy, commonly used in small-sample neurofeedback and neuromodulation studies^36,37^, allows assessment of overall effects while examining outcomes specifically in those who achieved the intended neural modulation. In the all-participants analysis, all four FS metrics improved significantly (steps per second, mean peak velocity, acceleration magnitude, and shorter step intervals), whereas in the responders-only analysis, both speed metrics remained significant and the other two showed the same direction of change, though with wider confidence intervals (Fig. 4). Together, these findings indicate that the behavioral effects are consistent across analyses and not driven by any single participant, and that the minor loss of significance with reduced sample size reflects the expected limitation of statistical power rather than a reversal of the effect^38^. Mental strategies varied across individuals between motor-related imagery and non-motor approaches. Previous studies have shown that motor imagery is particularly effective for engaging sensorimotor circuits and reducing beta synchrony^39–41^. The success of non-motor strategies such as focused attention or relaxation suggests that multiple cognitive approaches may support beta downregulation, emphasizing the need for flexible strategies during neurofeedback training^42^.

While beta-power could be reduced in the neurofeedback training, it was not significantly reduced compared to the baseline rest condition in the motor task blocks. This was unexpected, as voluntary movement is typically accompanied by beta desynchronization in the STN^4^. A potential explanation could be that preceding neurofeedback blocks may have had lingering effects on baseline beta activity. Moreover, participants were tested on medication and under ongoing DBS stimulation, both of which are known to suppress pathological beta activity^6,8,43^. These treatment effects may have led to already reduced beta-power during baseline rest, thereby limiting the observable range for further neurofeedback-induced suppression during motor execution. In other words, if beta activity was already attenuated at rest, additional downregulation during movement may have reached a physiological floor effect. Additionally, beta-power downregulation was less successful when participants attempted it between the two motor tasks (pre and post), particularly during the HPS blocks. This attenuation may reflect increased cognitive load, motor interference, or fatigue^44,45^ and highlights the importance of decoupling neurofeedback and motor phases in future designs^15,17^. Notably, Bichsel et al.^46^ demonstrated that externally cued (goal-directed) motor tasks are accompanied by event-related beta desynchronization, whereas self-paced (habitual) movements exhibit tonic beta suppression without clear event-locking. These distinct patterns suggest that goal-directed tasks require greater cognitive engagement, which may compete with the cognitive resources needed for effective self-regulation during neurofeedback. Together, these findings underscore that the context and timing of neurofeedback delivery - especially its interaction with ongoing motor and cognitive demands - play a critical role in modulating its effectiveness.

Previous work has employed wearable sensors to analyze gait^27,28,47^ or tremor^26,29,48^ in PD, and quantitative methods for evaluating leg agility (MDS-UPDRS 3.8)^30,49^ and hand pronation-supination (MDS-UPDRS 3.6)^24,50^ have been explored, however, their application in evaluating therapeutic interventions remains limited^51^. The two tasks were selected because they provide controlled, reproducible conditions that are particularly sensitive to short-term motor fluctuations. Clinically, impairments of hand function are among the most common deficits in PD and show strong correlations with overall motor disability, making improvements in these domains both precisely measurable and highly relevant to patient functioning^52,53^. By contrast, items such as gait and posture, although clinically important, are more variable, require larger testing environments, and are less suited for precise and reproducible quantification in a single-session IMU-based design^27,28,47,51^. Furthermore, the selected tasks can be performed in seated position and require less cognitive load than, e.g. gait or posture control, and are easier to focus on, while the risks of falls is mitigated. For these reasons, the two tasks were included as standardized alternatives to capture movement quality. This approach enables objective and reproducible analysis of motor improvements, particularly focusing on movement speed and amplitude. It is especially relevant for detecting subtle therapeutic effects, which may not always be apparent in conventional clinician-rated MDS-UPDRS scores, thereby enhancing the standardization of motor evaluations in PD.

After neurofeedback-induced beta-power downregulation, we observed improvements in lower limb movement quality. Specifically, FS post-neurofeedback showed significant increases in acceleration magnitude and movement speed, along with a reduction in halts. These findings are consistent with prior studies linking decreased beta-power to enhanced movement initiation, speed and performance^4,54,55^. Importantly, IMU-based metrics allowed for detecting subtle, short-term motor changes that may be missed by standard clinical observation. Two effects should be distinguished: (i) beta-power levels tended to correlate negatively with FS speed, whereas (ii) neurofeedback-induced beta reductions robustly predicted within-subject speed gains. This indicates that it was not merely lower beta-power per se, but specifically its modulation through neurofeedback, that was linked to behavioral improvement. Notably, the association between beta reduction and speed gain was remarkably strong, indicating that participants who achieved greater neural modulation also showed larger behavioral improvements. This close coupling between physiological and behavioral change supports the mechanistic link between beta modulation and motor performance.

The effect size analysis further supports these findings: FS metrics such as steps per second and mean peak velocity showed large improvements, indicating that the observed changes were not only statistically significant but also practically meaningful. In contrast, other measures (e.g., decrementing slope and amplitude) yielded small or negligible effects, suggesting limited or inconsistent changes. Importantly, although many confidence intervals included zero, the direction and magnitude of the effects suggest clinically relevant improvements, particularly in FS motor tasks.

While the present findings suggest that DBS-based neurofeedback contributed to these improvements in movement quality, other factors–such as intraindividual variability in motor performance or non-specific effects of repeated task execution–may also have played a role. However, the short time interval between pre- and post-assessments, the participants’ prior familiarity with the tasks, the generally high test-retest reliability of the MDS-UPDRS III assessment^56^, and the temporal alignment of improvements with beta-power reductions support the interpretation that neurofeedback was a key contributor.

The possibility of test–retest (practice) effects was also considered, since tasks were repeated within a short time frame. Statistical comparisons between baseline and pre-neurofeedback motor tasks revealed significant changes in only three variables of hand and foot motor tasks (FS mean peak velocity, FS acceleration magnitude, and HPS turn intervals), all of which showed worse performance at pre-neurofeedback compared to baseline. This indicates that simple repetition did not yield performance improvements, supporting the interpretation that the post-neurofeedback effects observed were not driven by test–retest influences but more likely linked to neurofeedback-induced beta-power modulation.

lower limb dysfunction in PD is a key contributor to mobility limitations and fall risk^57,58^. Our findings suggest that IMU-based assessments can detect motor improvements following neurofeedback, even when changes on conventional clinical scales are not visible. These metrics may serve as early indicators of therapeutic efficacy and support personalized rehabilitation. However, the clinical relevance of the observed improvements remains to be confirmed. The absence of clinician-rated MDS-UPDRS scores for items 3.8 and 3.6 at pre- and post-neurofeedback prevents direct comparison with conventional measures and determination whether the observed changes meet the threshold for clinically meaningful improvement. However, the inclusion of standardized effect sizes helps to describe the magnitude of change beyond p-values, offering a standardized metric for interpreting practical relevance, as large effect sizes observed in foot-stomping speed metrics, suggest changes that may approach the range considered meaningful in gait-related PD interventions.

While previous studies have explored neurofeedback in conjunction with hand motor tasks^15,22^, the HPS task in the present study did not show significant post-neurofeedback changes. This discrepancy likely reflects a combination of task- and circuit-specific differences in beta modulation. FS is a repetitive, impact-based task with more pronounced kinetic features, making it more accessible to IMU-based change detection, whereas HPS involves finer, cognitively demanding wrist rotations that may require longer or more targeted neurofeedback training to yield measurable effects^24^. The fixed order of task presentation may have further introduced mental fatigue, as HPS was always performed after FS at the end of the session.

Although upper limb movements can show strong dopaminergic and STN-related modulation^37,59–61^, this strongly depends on task characteristics. Complex, goal-directed actions such as reaching or grasping typically evoke stronger beta desynchronization than repetitive pronation–supination, which may elicit weaker or inconsistent modulation^62^. Notably, a previous study using a similar pronation–supination task reported significant modulation^24^, suggesting that factors such as the fixed task order, shorter training duration, or fatigue toward the end of the session in the present study may have attenuated the observable effect. In contrast, gait and foot-stomping tasks reliably engage beta dynamics^63^, consistent with the present lower limb findings.

Physiological and anatomical factors may further contribute. While leg movements are associated with stronger desynchronization in higher beta bands (24–31 Hz) compared to upper limb movements, indicating some limb-specific spectral specialization within the STN^64^, but upper limb modulation has also been shown in similar tasks^24^. The lack of such effects here may instead reflect contextual factors such as task order, shorter training duration, or fatigue. Moreover, STN neurons exhibit heterogeneous encoding of limb activity, with some units preferentially responsive to lower limb movements^65^. The fine motor demands, attentional load, and biomechanical complexity of the HPS task might further reduce the signal-to-noise ratio of feedback-driven modulation, consistent with evidence that cortico-kinematic coupling degrades in more complex movements^66^. Heterogeneity within the beta range (e.g., low vs. high beta) may further confine upper limb modulation to narrower sub-bands that fall outside the 5 Hz feedback window used^67^.. In addition, testing in the ON-medication state with ongoing DBS–both known to attenuate pathological beta–may have reduced the residual capacity for further modulation, particularly in upper limb circuits. Electrode placement variability could also have weakened upper limb beta coupling^68^. The lack of HPS effects, confirmed by equivalence testing, therefore most likely reflects limited task sensitivity and physiological ceiling effects rather than a failure of the neurofeedback approach itself.

Finally, exploratory correlations between limb-specific MDS-UPDRS III subscores and beta-power downregulation strength revealed no significant associations, although moderate negative trends were observed in the OFF state, particularly for hand pronation–supination. These findings suggest that baseline limb impairment did not systematically influence the ability to volitionally suppress subthalamic beta-power in this small cohort.

This study has several limitations. Beta-power downregulation was not statistically significant during the neurofeedback phases embedded within the motor task blocks, though a small visible reduction was observed, particularly in the FS condition. This may reflect limited attentional capacity during movement preparation or a floor effect from prior downregulation. It suggests that neurofeedback may be more effective when delivered in separate, focused blocks rather than interleaved with motor tasks.

Furthermore, the small sample size (8 responders out of 10 participants) reduces statistical power and limits generalizability. Nonetheless, small cohorts, for example a range of 3 - 19 participants^15,18,22^ are common in Parkinson’s disease neurofeedback research due to the challenges of recruiting patients with implanted DBS systems, but still, prior studies with comparable sample sizes have provided valuable insights. Importantly, the classification of two participants as non-responders underscores interindividual differences in responsiveness, a factor that requires further investigation.

Another limitation of this study is the lack of standardized information on the timing of medication intake relative to the neurofeedback session. All participants were tested in the ON-medication state, but fluctuations in motor performance and beta activity are strongly influenced by the pharmacodynamics of dopaminergic treatment. In particular, levodopa has been reported to exert stronger effects on upper limb function than on lower limb function^60,69^, which may have contributed to a potential ceiling effect in the upper limb and blunted the measurable impact of neurofeedback. Because the exact time since last dose was not systematically recorded, medication-related variability as a confounding factor cannot be excluded. Future studies should therefore adopt standardized ON testing conditions (e.g., consistently 60–90 minutes post-dose) or include OFF-medication sessions to better disentangle medication and neurofeedback effects.

While electrode contacts were clinically selected to target the dorsolateral STN and recordings were taken from adjacent bipolar channels, precise localization relative to STN somatotopy was not available. Given the medial-lateral organization of leg and arm representations^70,71^, spatial factors could plausibly influence both responsiveness and limb-specific effects. Incorporating imaging-based electrode localization in future studies may help clarify the anatomical specificity of neurofeedback effects.

The study was limited to a single session without repeated assessments over time or continuous monitoring. This restricts the ability to evaluate the stability and persistence of the observed effects, as well as their relevance to everyday motor performance.

Additionally, the fixed task order could have introduced mental fatigue effects, particularly for HPS. Finally, while IMU-based metrics detected subtle improvements, their validation in clinical settings remains to be established.

Future studies should test longer and repeated neurofeedback sessions, addressing individual variability, to learn how much training is needed for lasting benefits and relevance to everyday movement quality. Objective metrics for movement quality, as developed in this study, should be refined and validated across larger populations and using control mechanisms like motion capture and clinician ratings to investigate accuracy and temporal resolution, and to standardize motor task evaluations. Validating these metrics will require a separate dataset, which should be addressed in future work. Such a dataset should include clinician-rated MDS-UPDRS scores, test–retest assessments, and a sufficiently large sample size.

This study provides evidence that single-session DBS-based neurofeedback can lead to measurable improvements in motor performance in individuals with PD, particularly in lower limb function. By employing MDS-UPDRS III aligned metrics derived from wearable IMUs, we captured subtle motor changes often undetected by conventional clinical assessments, including speed, acceleration, and halts. These findings suggest that neurofeedback could evolve into a clinically relevant adjunct therapy, complementing medication and DBS programming, especially in domains where these conventional therapies provide limited benefit.

## Methods

### Participants and ethical considerations

Ten individuals with idiopathic PD were recruited within three weeks after STN-DBS implantation at the University Hospital Zurich. The sample size was based on previous power calculations, which has shown that a sample size of 6 participants is sufficient for finding a significant difference between baseline and neurofeedback regulation, based on a significance level of alpha = 0.05/2, and statistical power of 0.8^72^. The recording time window was chosen to allow performing the experiment in the structured clinical environment, and less influence of DBS due to the lower stimulation amplitude, which is typically increased over the months following implantation. Recordings took place during their rehabilitation at Klinik Lengg. All participants were right-handed and met inclusion criteria: age $\ge$18, clinical indication for Percept^™^ PC neurostimulator implantation, and subsequent planned hospitalization of at least three days. Exclusion criteria included reduced life expectancy (<1 year), impaired consciousness (GCS <15), communication difficulties, significant comorbidities, inability to follow procedures, insufficient language skills, or inability to provide informed consent. All participants provided written informed consent and were assigned anonymized identifiers. The study protocol was approved by the Cantonal Ethics Commission Zurich (BASEC 2021-00352) and conducted in accordance with the Declaration of Helsinki and local regulations. Data were encrypted at the point of acquisition, and only authorized personnel under confidentiality agreements had access to identifiable information. Medication and DBS settings remained unchanged throughout the study. All participants were tested in their ON-medication state. However, the exact timing of the last medication intake prior to the session was not systematically recorded. Participant demographics and session-related metadata are summarized in Table 3.

**Table 3:** Participant metadata. **LED**: Levodopa Equivalent Dose. **IMU**: Inertial Measurement Unit. **MDS-UPDRS III**: Movement Disorder Society Unified Parkinson’s Disease Rating Scale III. **NF:** Neurofeedback.

**PID**	**Sex**	**Age [y]**	**LED [mg]**	**Years since diagnosis**	**Post-OP day of session**	**MDS-UPDRS III OFF/ON**	**STN recording side**	**Stimulation [mA]**	**Beta-peak**	**NF beta-power [Hz]**	**NF strategy**
01	F	41	750	3	19	18/9	left	0.9	19.53	17.03–22.03	finger tapping, walking
02	M	52	1010	8	13	18/11	right	0.9	27.34	24.84–29.84	woodwork, planing
03	F	71	1095	11	15	36/18	left	1.0	18.55	16.05–21.05	fist flexing, walking
04	M	69	1425	7	6	53/17	right	0.2	18.55	16.05–21.05	playing accordion
05	M	69	1375	13	14	40/28	right	0.8	25.39	22.89–27.89	fist flexing
06	F	62	1200	8	11	55/29	right	0.8	20.51	18.01–23.01	fist flexing
07	M	64	1315	6	19	44/27	right	1.1	25.39	22.89–27.89	fist flexing
08	M	55	865	8	16	78/24	right	0.9	26.37	23.87–28.87	cycling, dancing
09	F	40	705	9	14	59/14	left	0.9	26.37	23.87–28.87	fist flexing, breathing
10	M	73	900	5	14	30/17	right	0.7	20.51	18.01–23.01	meditation

### Study procedure

**Fig. 6:**
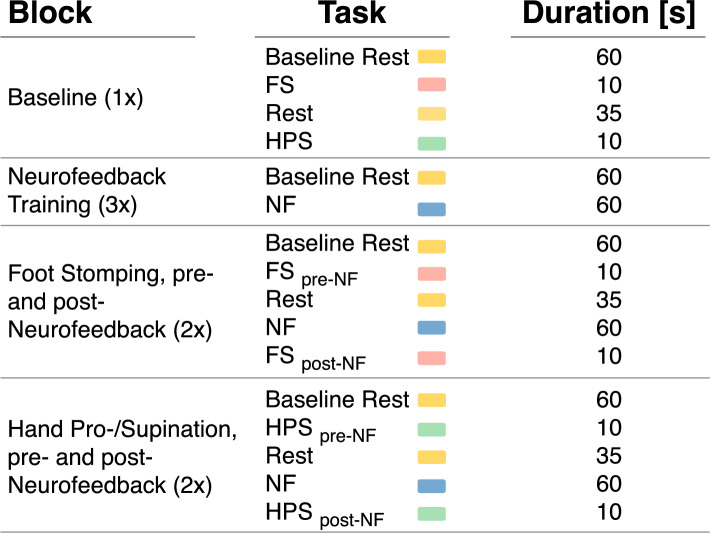
Study protocol. All participants underwent one session of 4 blocks. The number in brackets shows the number of block repetitions. Rest: Participants were instructed to sit quietly. NF: Neurofeedback downregulation of beta-power. FS: foot stomping (10x). HPS: Hand pronation-supination (10 cycles).

The study was conducted in a single session which lasted for about an hour comprising four measurement blocks (see Fig. 6). The first block served as a baseline, consisting of a 60-second rest period followed by baseline execution of the motor tasks (FS and HPS) without prior neurofeedback. A 35-second rest was inserted between tasks to minimize carryover effects.

Participants then completed three neurofeedback training blocks, containing a 60-second rest followed by a 60-second beta-power downregulation phase. During the downregulation phase, real-time visual feedback of subthalamic beta activity, referred to as neurofeedback, was displayed on a tablet. No feedback was shown during baseline rest and the motor tasks. To downregulate beta-power, the participants were encouraged to apply simple mental strategies (e.g., motor imagery of hand or walking movements) and could adjust their approach throughout. Reported strategies are summarized in Table 3.

Following training, FS and HPS were each repeated in dedicated post-training blocks to assess changes in movement quality. Overall, each movement block therefore consisted of: (i) a 60-second rest, (ii) a pre-neurofeedback motor task, (iii) a second rest, (iv) a 60-second neurofeedback session, and (v) a post-neurofeedback motor task. This sequence was conducted separately and in fixed order for FS (lower limb) and HPS (upper limb).

### Neurofeedback

The neurofeedback procedure was adapted from a previous study^21^. LFPs were recorded from the STN contralateral to the symptom-dominant side using Medtronic SenSight™DBS leads (B33005) connected to a Percept™PC neurostimulator (B35200). Each participant’s beta-peak frequency was determined with BrainSense™Survey before starting the protocol, allowing for individualized feedback. All participants were set up with monopolar stimulation, so that bipolar LFP recordings were configured with BrainSense™Setup using contacts adjacent to the active stimulating electrode. The latter was surgically identified to provide optimal coverage for dorsolateral STN. Across participants, the selected contacts for beta-power monitoring were consistently centered around contacts 2 and 10 (left/right). Beta-power was computed as the average power within a 5 Hz band centered on the beta-peak. Stimulation remained active throughout all recordings as part of routine clinical care.

Visual neurofeedback was delivered via the BrainSense™Streaming function on the Medtronic clinician programmer tablet. The interface updated beta-power measurements at a 2 Hz rate, displaying both the most recent 6-second window and the entire session. Participants were instructed to concentrate on reducing the beta-power in the highlighted 6-second window, which provided immediate performance feedback.

### DBS data processing and analysis

LFP data were exported in JSON format from BrainSense™ and analyzed using Python 3.9. Recordings were segmented according to task durations (see Fig. 6), and task-specific data from repeated motor blocks (FS and HPS) were averaged across both repetitions. To validate beta-peak selection, the power spectra of baseline rest data were plotted (Fig. 2).

Changes in beta-power across conditions were assessed using BrainSenseLfp data (i.e., power in a 5 Hz window around the beta-peak, sampled at 2 Hz). Each block was normalized to the median of its initial rest period. Statistical comparisons were performed using Wilcoxon signed-rank tests, following Shapiro–Wilk tests for normality. TFRs of the BrainSenseTimeDomain signal were computed using complex Morlet wavelets (cmor0.5–5.0, via PyWavelets^73^, adapted from^54^). A Savitzky–Golay filter (length 20, order 3) was applied to smooth high-frequency noise and improve visualization. The resulting TFRs enabled a qualitative comparison of beta-band modulation across tasks (Fig. 3). Participants were classified as responders or non-responders based on their ability to modulate beta-power during neurofeedback: responders showed a reduction in beta-power in the third neurofeedback training block, while non-responders displayed increased beta-power after three neurofeedback trainings (P01 and P09).

### Handling of non-responders

Non-response to neurofeedback is a recognized phenomenon and should be monitored and reported. Responders were defined a priori as participants who downregulated STN beta-power during neurofeedback (third training block < baseline); while non-responders could not achieve beta downregulation. Two analysis sets were prespecified: 1) all participants (N=10): representing a conservative intention-to-treat analysis; and 2) responders only (N=8), to examine behavioral effects specifically in those who successfully modulated beta-power. Additionally, the effect of excluding each non-responder (P01, P09) separately, as well as both together was tested using paired comparisons with Wilcoxon signed-rank tests. Effect sizes are reported as standardized mean differences (SMD) with bootstrapped 95% CIs.

### Motor tasks

To assess changes in movement quality, two standardized MDS-UPDRS III tasks were selected, targeting the upper and lower limb. FS, based on item 3.8 (Leg Agility), evaluated lower limb function. Participants were instructed to perform ten seated, full-foot stomps as quickly and forcefully as possible. HPS, aligned with item 3.6 (Pronation-Supination Movements of Hands), assessed upper limb function through ten rapid, alternating wrist rotations, aiming for maximum range and speed.

Tasks were executed with the limb on the symptom-dominant side, contralateral to the STN recording site (see Table 3). To enable objective motor task assessment, inertial measurement units (ZurichMove®) were placed on the ankle (FS) and wrist (HPS), capturing triaxial acceleration, angular velocity, and orientation at 200 Hz. Sensors were aligned to match the main axis of motion for each task.

### Movement quality metrics

Movement quality was defined as a set of quantifiable features reflecting the performance of motor tasks, including amplitude, speed, smoothness, and rhythmicity. Task-specific metrics were derived from IMU data, aligned with MDS-UPDRS III criteria. IMU signals were manually segmented into task-specific time windows by visually identifying periods of active movement. Segmented data were downsampled to 50 Hz prior to further analysis.

*Foot Stomping (FS)*: Preprocessing of FS accelerometer data included: (i) transformation from the sensor to global frame using ZurichMove® quaternions, (ii) conversion from g to m/s^2^, (iii) gravity compensation (9.81m/s^2^ subtracted from the z-axis), and (iv) high-pass filtering at 0.5Hz to remove low-frequency drift. To derive velocity and distance, the processed acceleration data were integrated twice using a zero-update approach to limit drift. A peak detection algorithm identified foot strikes, verified visually, to identify movement segments. Velocity was computed by integrating within each movement segment, followed by drift correction. A second integration yielded displacement estimates.

*Hand Pronation-Supination (HPS):* Gyroscope data, recorded in $^{\circ }$/s, were used for HPS as the angular velocity directly records the turning motion of the wrist. As the signal was smoother, only quaternion-based frame transformation and 0.5 Hz high-pass filtering were applied. Angular displacement (in degrees) was obtained via single integration; no zero-update was applied due to sufficient signal stability.

For both tasks, kinematic metrics such as magnitude, speed, amplitude, halts, smoothness, and decrementing amplitude were derived from IMU data. Where available, published definitions were applied - particularly for smoothness - while custom metrics were developed to align with the scoring logic of the MDS-UPDRS III. These features were computed from velocity and displacement (accelerometer data) or angular displacement (gyroscope data), based on established kinematic principles. All metrics were stored in a structured data frame and compared between pre- and post-neurofeedback using the Wilcoxon signed-rank test, as well as the SMD including confidence intervals to measure effect size. The results are reported in Table 2.

To assess potential test–retest effects, baseline motor tasks metrics (obtained before the neurofeedback administration) were additionally compared with pre-neurofeedback metrics using the Wilcoxon signed-rank test. Detailed definitions and computational approaches are provided below.

**General Metrics:** The acceleration or angular velocity magnitude was defined as $\sqrt{x^2 + y^2 + z^2}$, where $x, y, z$ are the three IMU axes. This follows the formula described in^30^.

**Speed:** Step or turn rates were computed as the number of peaks per second, $\text {Rate} = N_{\text {peaks}} / T$, where $N_{\text {peaks}}$ is the number of detected peaks and $T$ is the segment duration in seconds.

Mean peak velocity (FS) and angular velocity (HPS) were calculated as the average of the mean peak values across movements: $\text {MeanPeak} = (1/N) \sum _{i=1}^{N} v_{\text {peak}, i}$, where $v_{\text {peak}, i}$ is the peak velocity of movement $i$.

**Amplitude:** Mean amplitude was determined as $\text {MeanAmplitude} = (1/N) \sum _{i=1}^{N} A_i$, where $A_i$ is the amplitude of movement $i$.

Total displacement was computed as $\text {TotalDisplacement} = \sum _{i=2}^{N} |x_i - x_{i-1}|$, with $x_i$ denoting the displacement at time $i$.

**Hesitations:** Hesitations were defined as movement smoothness, quantified using spectral arc length, following^20^.

In addition, the log-dimensionless jerk was estimated from the velocity signal as described by^74^.

**Halts:** Step or turn intervals were computed as $\text {MeanInterval} = (1 / (N - 1)) \sum _{i=2}^{N} (t_i - t_{i-1})$, where $t_i$ is the timestamp of movement $i$. Halts were identified as intervals exceeding two standard deviations above the mean: $\text {Halts} = \sum _{i=2}^{N} \textbf{1}_{(t_i - t_{i-1})> \mu + 2\sigma }$, with $\mu$ and $\sigma$ being the mean and standard deviation of all intervals.

**Decrementing Amplitude:** Change in amplitude over time was defined as $\Delta A = \bar{A}_{\text {first}} - \bar{A}_{\text {second}}$. The slope of amplitude across time was estimated via linear regression $A(t) = mt + b$, where $m$ represents the rate of change over time.

### Analysis of beta-power and movement quality relationship

LFP and IMU data streams were not directly synchronized in real time; instead, motor task periods were retrospectively identified from IMU recordings using algorithmic segmentation and aligned with the fixed timing of the study protocol. To explore whether beta-power downregulation was associated with changes in motor performance, we computed Pearson’s correlation and linear regression for significantly improved FS metrics. Both beta-power and movement metric were z-scored across all trials to standardize effect sizes. Each participant contributed five FS trials: one during baseline, two during pre-neurofeedback, and two during post-neurofeedback. We further tested whether task phase (baseline, pre-neurofeedback, post-neurofeedback) moderated the relationship by including it as a categorical factor in the regression model.

To disentangle level from downregulation effects of beta-power, two complementary correlation analyses were performed. First, a baseline-only correlation was computed between z-scored beta-power during the first baseline FS and z-scored FS speed (steps per second) across participants. Second, a modulation analysis was conducted using change scores: $\Delta \beta = \beta _{pre-NF}-\beta _{post-NF}$ (larger values reflect greater beta reduction) and $\Delta \textrm{speed} = \textrm{speed}_{\mathrm {post\text {-}NF}} - \textrm{speed}_{\mathrm {pre\text {-}NF}}$. For each participant, $\Delta \beta$ and $\Delta \textrm{speed}$ were computed and correlated using Spearman–s $\rho$ with bootstrap confidence intervals (5000 resamples). Analyses were performed for all participants and for responders-only.

To further assess the absence of upper limb effects, a paired equivalence test (two one-sided tests with smallest effect size of interest ±0.3 SD of the pre value) was performed for the HPS mean peak velocity metric to evaluate whether pre–post changes were statistically equivalent to no meaningful effect.

In addition, it was explored whether the ability to downregulate beta-power related to baseline limb-specific motor impairment. For each patient, neurofeedback effect strength was defined as the relative reduction in normalized LFP beta-power during “down” periods compared to baseline, averaged across blocks, and analyzed separately for FS and HPS. Baseline impairment was quantified from preoperative MDS-UPDRS III items 3.8 (leg agility) and 3.6 (hand pronation-supination), using the score of the limb side measured in the study. Spearman correlations were calculated for both OFF and ON medication states.

## Supplementary Information

Supplementary Information.

## Data Availability

The datasets generated and analysed during the current study are available from the corresponding author upon reasonable request.
