# Supplementary material for: Lower limb motor effects of DBS neurofeedback in Parkinson’s disease assessed through IMU-based UPDRS movement quality metrics
Source: Sci Rep. 2025 Nov 27;15:44579. doi: 10.1038/s41598-025-28378-8 (PMC12738743; doi:10.1038/s41598-025-28378-8)
Supplement: Supplementary file 1 — Supplementary Information. [file 41598_2025_28378_MOESM1_ESM.docx]

Figure S1 Normalized beta-power during pre-and post-neurofeedback (NF) motor task blocks for responders (N=8). (a) Foot Stomping (FS) and (b) Hand Pronation-Supination (HPS) conditions. Each participant is represented by a unique marker. Boxplots show median and interquartile range of beta-power normalized to the baseline rest period (dashed line). While beta-power tended to decrease during NF and post-NF in FS, no consistent modulation was observed for HPS.
